# Antileishmanial metallodrugs and the elucidation of new drug targets linked to post-translational modifications machinery: pitfalls and progress

**DOI:** 10.1590/0074-02760220403

**Published:** 2022-03-23

**Authors:** Rubens Lima do Monte, Paulo Otávio Lourenço Moreira, Alessandra Mara de Sousa, Miguel Antonio do Nascimento Garcia, Suellen Rodrigues Maran, Nilmar Silvio Moretti

**Affiliations:** 1 Fundação Oswaldo Cruz-Fiocruz, Instituto René Rachou, Grupo de Pesquisas em Biotecnologia Aplicada ao Estudo de Patógenos, Belo Horizonte, MG, Brasil; 2 Universidade Federal de São Paulo, Departamento de Microbiologia, Imunologia e Parasitologia, Laboratório de Biologia Molecular de Patógenos, São Paulo, SP, Brasil

**Keywords:** Leishmania, metallodrugs, antileishmanial, target elucidation, post-translational modifications

## Abstract

Despite the increasing number of manuscripts describing potential alternative antileishmanial compounds, little is advancing on translating these knowledges to new products to treat leishmaniasis. This is in part due to the lack of standardisations during pre-clinical drug discovery stage and also depends on the alignment of goals among universities/research centers, government and pharmaceutical industry. Inspired or not by drug repurposing, metal-based antileishmanial drugs represent a class that deserves more attention on its use for leishmaniasis chemotherapy. Together with new chemical entities, progresses have been made on the knowledge of parasite-specific drug targets specially after using CRISPR/Cas system for functional studies. In this regard, *Leishmania* parasites undergoe post-translational modification as key regulators in several cellular processes, which represents an entire new field for drug target elucidation, once this is poorly explored. This perspective review describes the advances on antileishmanial metallodrugs and the elucidation of drug targets based on post-translational modifications, highlighting the limitations on the drug discovery/development process and suggesting standardisations focused on products addressed to who need it most.

Historical aspects and recent advances on antileishmanial metallodrugs

The growing interest on developing bioactive compounds containing metals in their structure is due to the successful use of cisplatin in cancer chemotherapy,^1^ ^,^ ^2^ which inspired scientists to look for new metal-based drugs for parasitic diseases such as leishmaniases. Indeed, the metalloid antimony (Sb^V^) is the active principle of the main antileishmanial drugs currently in clinical use to treat all forms of leishmaniases since 1940s; reviewed by Haldar et al.^3^ However, a significant increase in clinical resistance has been reported for this drug class in recent years.^4^ Complexes containing metals can expand the list of drug candidates due to the peculiar attributes concerning the presence of the metallic core add to the organic fragment.^5^ ^,^ ^6^ Metallodrugs can present unique properties due to the particular characteristics of metals, such as broad redox potential, coordination geometry, thermodynamic and kinetic properties, which can lead to an increase in the drug’s effectiveness, targeting and lipophilicity.^7^ ^,^ ^8^ Metal-based drugs are widely used in medicinal chemistry and their exploration relies on the fact they can be applied as covalent binders to biomolecules; as enzyme inhibitors; as redox-activating or photoactivatable compounds; as nanocarriers; as catalytic drugs; for imaging and radioactive therapy; reviewed by Boros et al.^9^ There is growing interest in metal-based drugs with potential application in a variety of therapeutic areas as they offer a rich source of effective chemotherapeutic agents especially when the drugs are selective towards specific targets. Here, we review recent advances on antileishmanial metallodrugs.

Although classified as a heavy metal, bismuth (Bi) shares several physicochemical properties with Sb and arsenic (As), both latter considered metalloids. Being the most nontoxic heavy element to humans, together with no evidence of resistant parasites, Bi became an attractive alternative.^10^ Several studies pointed out the antileishmanial effect of Bi, even in low micromolar range and against different *Leishmania* spp. like *L. major*, *L. amazonensis* and *L. infantum*, including Sb-resistant strains.^11^ ^,^ ^12^ Trivalent [Bi(III)] atoms were the most promising candidates with favorable selectivity. Despite of that, there is no advance on structure-activity relationship (SAR) studies and none of the complexes passed through *in vivo* efficacy assay; reviewed by Ong et al.^10^ On the other hand, transition metals, such as ruthenium [Ru(II)], promote programmed cell death providing hints on the mode of action (MoA) of Ru(II) against *L. amazonensis*.^11^ Upon complexation with the antiparasitic agent clotrimazol, ruthenium presents synergistic action.^12^ In this context, other metal ions like platinum [Pt(II)], copper [Cu(II)], rhodium [Rh(I)], osmium [Os(III)] have been used to obtain organometallic compounds with ligands derived from benzothiazole, a compound of which some derivaties have shown promising antiparasitic activity.^13^

Inspired by the repurposing potential of the gold-based antiarthritic drug auranofin (Fig. 1), several Au(I) and Au(III)-containing compounds are promising antileishmanial agents.^14^ ^,^ ^15^ ^,^ ^16^ ^,^ ^17^ ^,^ ^18^ The redox imbalance promoted by gold-based compounds, especially derived from Au(I) oxidation state, makes them good antileishmanial drugs. Trypanothione reductase (TR) is the most studied target for gold-based antileishmanial compounds. However, topoisomerase I, kDNA (kinetoplastid or mitochondrial DNA) and cysteine proteases are being proposed as gold targets, increasing the potential for drug selectivity; reviewed in Rosa et al.^18^ Several reports demonstrated *in vitro* activity against different *Leishmania* spp. at low micromolar range; reviewed by Rosa et al.,^18^ however, only two studies went through *in vivo* efficacy evaluation using experimental murine model, showing promising results with parasite burden reduction varying from 68 to 100% upon oral or IP (intraperitoneal) administration of 12.5 to 20 mg/kg/day of gold-based compound.^15^ ^,^ ^17^ For comparison, examples of metal-based drugs that underwent preclinical *in vivo* efficacy studies are presented in Fig. 1, as potential candidates to tackle leishmaniasis.

**Fig. 1:**
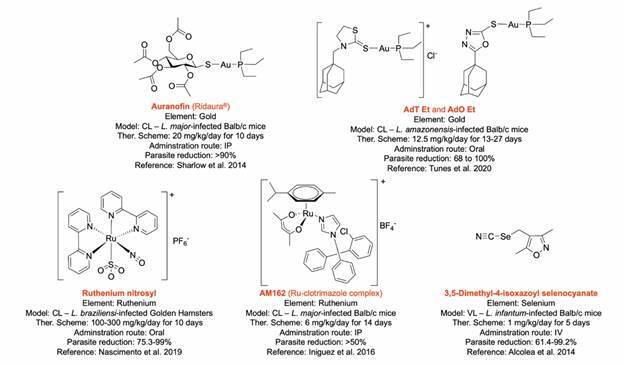
examples of metallodrugs presenting *in vivo* antileishmanial activity as potential drug candidates to tackle leishmaniasis. CL: cutaneous leishmaniasis; VL: visceral leishmaniasis; IP: intra-peritoneal; Ther: therapeutical. Molecular structures were reproduced from.^12^ ^,^ ^15^ ^,^ ^17^ ^,^ ^19^ ^,^ ^20^

Classified in the periodic table below gold (Au), silver (Ag) also displays good antimicrobial properties. When complexed with 1,10-phenanthroline-based ligands, Ag(I) complexes presented antileishmanial activity (against *L. amazonensis*) comparable to amphotericin B *in vitro*.^21^ Tetrahedral silver dppz (dipyridophenazine) or dpq (dipyridoquinoxaline) complexes showed leishmanicidal effect against *L. mexicana* and DNA affinity studies suggested they can tightly bind DNA.^22^

Like As and Sb, selenium (Se) is considered a metalloid and its antileishmanial activity has been explored *in vitro* against *L. donovani*. However, it requires high concentrations to inhibit the promastigote growth.^23^ Despite this fact, Alcolea and cols. (2021) showed that 3,5-dimethyl-4-isoxazoyl selenocyanate can reduce 99% of *L. infantum* parasite load in liver from visceral leishmaniasis (VL) murine model with low toxicity.^19^ Biogenic selenium nanoparticles also presented low micromolar action against *L. major in vitro* and prevented cutaneous leishmaniasis lesion caused by *L. major* in BALB/c mice at 5 mg/kg/day.^24^ Several metallic nanoparticles have been demonstrated to possess antileishmanial activity. High selectivity and efficacy against Sb-resistant/paromomycin-resistant *L. donovani* were achieved upon exposure to a quercetin conjugated gold nanoparticle.^25^ Silver (Ag0) nanoparticles are known to inhibit TR in *L. infantum*, being more effective than Sb, leading to antiproliferative effect.^26^ Titanium dioxide Ag nanoparticle (TiO_2_AgNps) combined with visible light exposure are promising alternatives to treat both VL and CL (Cutaneous Leishmaniasis).^27^ Magnesium oxide nanoparticles (MgONPs) were also associated with antileishmanial activity against *L. major* being able to silent cysteine protease b (*cpb*) and *gp63* coding genes.^28^

Like Au compounds, Ni(II) and Co(III) are transition metals that depending on its coordination and geometry diversity could also target TR leading to redox imbalance. When tested against *L. major* promastigotes, Ni(II) presented moderate activity,^29^ while in contrast, Co(III) complexed with *N*,*N*,*N’*-trisubstituted acylthiourea presented high activity with an IC_50_ value of 0.45 µM against *L. major*.^30^

VOSalphen, a vanadium (V) complex with stilbene derivative, presented an impressive *in vivo* efficacy in CL murine model due to *L. amazonensis* in BALB/c mice, upon intralesional administration of 75 µg/kg twice a week during 4 weeks.^31^

The aforementioned studies highlight the advances on the searching for metallodrugs against leishmaniasis. However, the lack of protocol standardisation on the use of animal models, administration route, experimental leishmaniasis-causing *Leishmania* spp., dose, therapeutical scheme, toxicological and drug combination approaches are limiting factors that impact study comparison and the advance in the field. Indeed, despite the increasing number of reports highlighting the importance of antileishmanial metallodrugs *in vitro*, very little goes further in the drug development pipeline.

Challenges on drug discovery/development pipeline for antileishmanial metallodrugs

Target identification/validation would be the ideal beginning of the drug discovery workflow. However, several academic-based studies start from empirical approaches guided by a trial and error basis. Drug discovery and development are essentially by origin a multidisciplinary area were chemists, pharmacists, physicists, biologist, computer scientists, and so on, must work together even from the beginning delineating the rationale that will guide the whole process. Academic-focused projects within a context of limited resources where research groups are evaluated by the number of published papers and note necessary quality, will hardly pursue through the next steps. The growing number of manuscripts showing new chemical entities with potential antileishmanial property does not follow the emergence of new products to treat leishmaniasis, and the reason for that is a huge gap between academic research and the industry interest, passing through the economical politics from the State to save people from suffering of neglected tropical diseases, commonly associated to poverty. In this regard, the Drugs for Neglected Diseases *Initiative* (DND*i*), a not-for-profit organisation is trying to reduce this gap in order to provide treatments to who need it most. But to achieve this aim, collaborative work and standardisation of protocols are required. DNDi have proposed the term target candidate profile (TCP) in order to standardise the minimum requirements for lead optimisation and drug development to treat leishmaniasis (https://dndi.org/diseases/visceral-leishmaniasis/target-product-profile/). In this context, we suggest additional standardisations in the pre-clinical scenario of antileishmanial drug discovery. The following points should be carefully addressed when developing antileishmanial metallodrugs. A compilation of these steps can be found in Fig. 2.

**Fig. 2:**
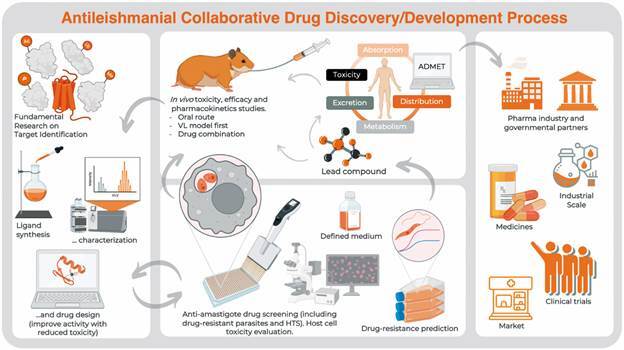
antileishmanial drug discovery and development pipeline. Target elucidation is part of fundamental research performed in universities and research centers. Drug target validation can be a result of collaborative work between pharmaceutical industry and academy. This initial step leads to the synthesis of new potential inhibitors that are synthesised and can be optimised after drug screening. Computer-aid strategies are helpful on rational drug design. Drug sensitivity values obtained during phenotypic screening on antileishmanial activity and drug toxicity are used to improve activity and reduce unwanted effects. Antileishmanial *in vitro* assays should be performed using intracellular amastigote forms. For that, the drug toxicity against the used host cell must be evaluated previously. Murine bone marrow-derived macrophages, murine peritoneal macrophages or human monocyte-derived THP-1 macrophages are commonly used as host-cells. When assaying metal-based drugs, it is important to choose defined or semi-defined culture medium, since reactive metal atom can interact with medium constituents and interfere with drug availability. The selection of resistant parasites by increased step-wise drug pressure can be an indicative of easy drug resistance acquisition. It can be included during *in vitro* phenotypic screening as an assay of drug-resistance prediction. Before moving forward through *in vivo* efficacy evaluation, it is strongly recommended to obtain the drug pharmacokinetic profile in a reduced group of golden hamsters. Drug administration preferable route is oral and visceral leishmaniasis model should be the first choice on evaluating systemic antileishmanial effect. If successful, cutaneous leishmaniasis model can be included. Drug combination is encouraged in order to prevent drug resistance emergence, increase efficacy and to reduce toxicity. *In vivo* efficacy and administration, distribution, metabolism, excretion and toxicity (ADMET) hints can feedback drug design and resynthesis. Predictive ADMET can also be assessed using computational approaches, like pkCSM tool (http://biosig.unimelb.edu.au/pkcsm).^34^ The crucial step on antileishmanial drug discovery/ development is to establish partnership with the pharmaceutical industry, especially during scaling up, production and for clinical trials. Consortia with government can afford legal and financial security. The gap between academic research and the industry against neglected tropical diseases (NTDs) will be reduced only after aligning goals and together with the private and public sectors. Parts of Fig. 2 were created with BioRender.com and are licensed under the agreement number: *JY23H65VZS*.

*Synthesis rationale* - A great amount of data highlighting antileishmanial compounds started by collaborations where the chemist designed a series of chemical products based on different reasons (e.g., novelty, synthesis route, physicochemical particular properties) but rationale must be based on pharmacophore studies. Target-based or ligand-based approaches can be considered previous to a new synthesis. It can also reduce time and costs of further molecule optimisation during drug development steps. Rationale-guided synthesis would save time, money and increases success rate.

*The yield* of an antileishmanial active compound and the complexity to obtain it must be included in this filtering equation. Sometimes this trial-and-error approach leads to an interesting compound, but there is no chance of enough quantity to pursue *in vivo* or more mechanistic assays. The group can meet the criteria for publishing a paper, but the contributions will be limited and the cycle tends to restart, generating “substrates” that cannot be translated into “products”.

*Computer-aid predictions* - This is a very powerful tool, following the advances of artificial intelligence and machine learning predictors. Although it can drive very good predictions for organic compounds, it could not be the same when considering organometallic molecules, since molecular docking and molecular dynamic predictions for metal atoms can be an additional challenge.

*High throughput drug screening (HTS)* - Several universities and research centers are able to collaborate with the pharmaceutical industry and perform HTS, mining drug candidates by fishing them based on phenotypic screenings. The limitation here is the filters required such as: solubility, one testing concentration, one target driven, stringent selectivity and potency. In this way, good candidates are filtered out. Sometimes, these rules are even incorporated in low or medium throughput screenings. There are several examples where selectivity index (SI) or IC_50_ did not discouraged an *in vivo* pilot PK (Pharmacokinetics) or efficacy studies.

*Culturing conditions* - To perform phenotypic screening against *Leishmania* parasites and to test cytotoxicity *in vitro* on mammalian cells, it is essential to pay attention on culture medium composition, since metallic compounds could interact with thiols or albumin, interfering with drug availability. Metallic compounds could also react with the milieu constituents, transforming into new species.^32^ Most compounds are solubilised in dimethylsulfoxide (DMSO) which can bind to metal atoms.

*Parasite evolutionary stage* - Despite the advantages of using promastigotes or axenic amastigotes for phenotypic screening, it is fundamental to include intracellular amastigotes, since metal-based compounds could act as prodrug where the presence of the host cell is essential for the mechanism of action. Drug-resistant parasites should also be included.

*Drug resistance prediction* - It is strongly encouraged to evaluate the potential for selecting drug-resistant parasites. Stepwise drug resistance selection can provide clues on predicting parasite’s adaptations towards drug tolerance or developing specific drug resistance mechanisms.^33^

*Drug combination* - Is also encouraged and justified by reducing the chances of selecting resistant parasites and to improve therapeutical schemes, reducing dose and treatment time.^17^ It could be tested *in vivo*, guided or not by previous *in vitro* isobologram tests.

*In vivo pharmacokinetic, toxicological and efficacy studies* - Pilot assays could save time and energy before continuing drug discovery pipelines. Minimal *in vivo* requirements should be achieved before systematically investigate safety and efficacy. Efficacy studies must consider the disease establishment prior to the treatment.

*Animal model and administration route* - The preferable administration route is oral and the *in vivo* VL model using golden hamsters is suggested as the first choice, since it provides more sensitive approach with systemic drug action.

Antileishmanial metallodrugs: product market fit

All currently available antileishmanial drugs are derived from repurposing strategy, including antimony-based compounds that were historically used for a plethora of applications, including cosmetics in ancient Egypt and to treat human trypanosomiasis in Africa; reviewed by Haldar et al.^3^ If antimony-based compounds were considered for current drug development pipelines it would probably be discontinued due to its toxicity. Recent technological advances allowed the use of nanotechnology to improve drug efficacy and reduce side effects. However, we should keep always in mind that neglected tropical diseases (NTDs) are related to poverty and high costs will prevent wide use. These features were considered by DND*i* when proposing the target product profile for antileishmanial drugs (https://dndi.org/diseases/cutaneous-leishmaniasis/target-product-profile/). Although the drug discovery process starts mostly at universities and research centers (Fig. 2), the final product should meet scalability for industrial production and affordable price. It could be counterintuitive, but gold-based therapy could cost less than Sb, miltefosine or amphotericin B treatments.^17^ Most countries suffering from leishmaniasis are poor with no access to treatments. Who pays the bill for treating NTDs? To answer that, we can be inspired by the global health solidarity during Coronavirus disease 2019 (COVID-19). Rich countries are protecting themselves when paying to treat NTDs in low-income economies led by a One Health approach and based on justice and equity, not only charity. As an example, Canada is importing *Leishmania*-infected dogs even not being endemic for leishmaniasis, which could start spreading the disease there, upon favorable conditions.^35^ So, why not help to eradicate it in a global effort empowering countries to circumvent NTDs? More effective and affordable treatments are urgent!

Progresses on targeting elucidation for antileishmanial drugs in the CRISPR/Cas9 and artificial intelligence (AI) era

The evidence used to validate drug targets can arise from genetic manipulations of a pathogen, such as by trying to obtain knockout/knockdown mutants of specific genes, which will give a clue about the gene essentiality/centrality and consequently would suggest the one as potential candidate for further studies as drug target. The process of discovering new targets with antileishmanial potential has undergone several changes in the last few decades with the establishment of new genetic engineering tools. During many years, the only tool available for gene replacement consisted of transfection of long linear constructs carrying a 0.5-1 kb long homology region, which significantly limited the success in obtaining gene deletion mutants in *Leishmania*. However, after the advent of an efficient CRISPR/Cas system in *Leishmania*,^36^ gene knockout was made easier, expanding our knowledge of parasite biology and the number of potential drug targets to be explored. Also, recent improvements on the method, for example, the association of Cas9 editing coupled with barcode-sequencing (bar-seq), or with the DiCre system, had made possible a high-throughput screening and a better validation of gene essentiality.^37^ ^,^ ^38^ Although we still face some difficulties to obtain knockout mutants using CRISPR/Cas9 for some specific targets and due to the intrinsic difficult to genetically manipulate some *Leishmania* species, such as *L. braziliensis*, the number of mutated genes raised from about 209 genes (before the CRISPR/Cas9 establishment) to about 400 genes nowadays.^39^ From these, it was observed that around 120 genes are essential for *Leishmania*, taking into account all parasite species (Fig. 3). Considering only genes related to post-translational regulation, the use of CRISPR/Cas9 tool contributed significantly to our understanding with numbers going from 25 genes before the establishment of this method to 268 genes after that (Fig. 3). These numbers will for certain increase dramatically once the recent establishment of the LeishGEM project (http://leishgem.org) led by Dr Jeremy Mottram (University of York) and Dr Eva Gluenz (University of Glasgow) is complete. LeishGEM will use CRISPR genome modification tools to generate 9000 *Leishmania* gene deletion mutants to determine which proteins are needed for the parasite to progress through its life cycle and survive in its host, opening the opportunity to soon explore these candidates as drug targets. Another revolutionary tool that will contribute enormously to drug target elucidation in *Leishmania* is the use of AI for protein structure prediction and ligand binding assays of specific compounds. Recently, the release by the DeepMind company of thousands of predicted protein structures from more than twenty species with high accuracy gained worldwide attention due to its potential to explore the AlphaFold tool in the drug target deconvolution.^40^ The whole predicted proteome of *L. infantum*, including most of the enzymes involved in post-translational modification (PTMs) regulation, was released and will help us to advance in the development of antileishmanial drugs focused in this mechanism.

**Fig. 3:**
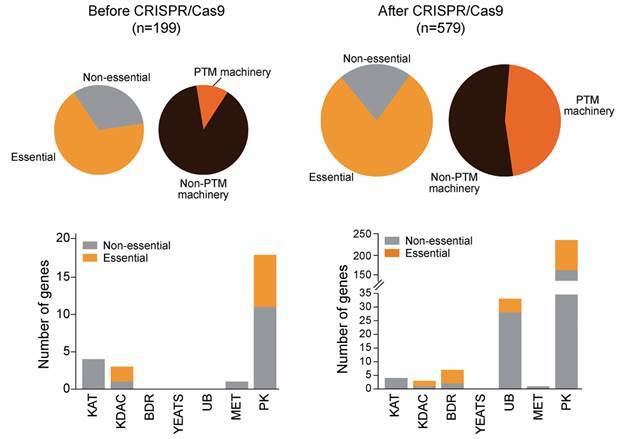
impact of CRISPR/Cas9 in the identification of potential drug targets in *Leishmania* related to post-translational modification (PTMs). The number of *Leishmania* genes with published attempts at the creation of a null mutant almost tripled after the establishment of CRISPR/Cas9 technology compared to the period where the main method consisted in the homologous recombination of fragments bearing homology regions alone (upper panel). This is more evident for *Leishmania* genes belonging to PTM regulatory machinery (upper panel, right data), and especially for those involved in the regulation of phosphorylation (PKs), ubiquitination (UB) and acetylation (BDR) (lower panels).

Drug target validation and the prospection of new antileishmanial candidates based on post-translational regulatory machinery

Environmental adaptation is regulated at different levels, from gene expression to enzymatic strategies. Reversible PTMs represent a relatively fast method to control protein activity, enabling more responsive cellular actions. Multiple PTMs, including phosphorylation, ubiquitination, methylation, crotonylation, acetylation^39^ ^,^ ^41^ ^,^ ^42^ ^,^ ^43^ ^,^ ^44^ ^,^ ^45^ are important to orchestrating the cellular transformations that occur as the *Leishmania* parasite shifts between its invertebrate and vertebrate hosts. The regulation of PTMs levels is made possible by the action of a group of enzymes with activities against specific amino acid residues in particular proteins. The advent of highly sensitive proteomic approaches revealed the presence of a myriad of PTMs present in thousands of proteins involved in key cellular processes in trypanosomatids.^46^ ^,^ ^47^ ^,^ ^48^ ^,^ ^49^ Associated with that, preliminary characterisation of some of the enzymes involved in the regulation of these PTMs suggests them as potential candidates to be explored as drug targets in these parasites. In the next sections we will give an overview of how the enzymes involved in PTMs regulation have the potential to be explored as drug targets in *Leishmania*.

*Protein phosphorylation* - Phosphorylation is arguably the most pervasive and best-studied PTM, and intimately associated in almost every cellular process. It plays key roles in modulating the activities of numerous enzymes by regulating protein-protein interactions or by changing protein conformation. This is achieved through the covalent attachment of phosphate groups to serine, threonine, and tyrosine residues by protein kinases, and their removal by protein phosphatases.^50^ ^,^ ^51^ Several changes in the phosphorylation profile were observed among the different *Leishmania* parasite stages, confirming the importance of this modification during parasite adaptation throughout their life cycle,^46^ ^,^ ^47^ ^,^ ^52^ and opening the opportunity to explore the repertoire of kinases and phosphatases as drug targets, as has been done extensively for the treatment of a variety of diseases in humans.^53^ The *Leishmania* kinome has 195 eukaryotic protein kinases (ePKs) that are classified according to catalytic domain conservation in six groups (CMGC, AGC, CAMK, STE, NEK, CK1). Additionally, atypical kinases, which lack a conserved ePK structure, but are catalytically active, are present in *Leishmania*.^38^ ^,^ ^54^ ^,^ ^55^ Recently, Baker et al., using high-throughput application of Cas9 editing associated with bar-seq investigated the functional importance of the whole *L. mexicana* kinome at key developmental stages of the parasite, revealing 44 protein kinases (PKs) essential for procyclic stage, 15 PKs important for procyclic to metacyclic differentiation in the vector and 29 PKs that are crucial for the parasite development in the mammalian host.^38^ The PKs have been explored as drug targets in the last decades in *Leishmania*.^56^ ^,^ ^57^ Indeed, a cyclin-dependent kinase 12 (CRK12) inhibitor is currently undergoing clinical studies as an antileishmanial drug,^58^ and with the new insights provided by all the efforts currently employed using the new genetic manipulation tools several more PKs are in the spotlight with the potential to be explored for the development of new chemical antileishmanial compounds soon.

*Protein acetylation* - Protein acetylation occurs by the addition of an acetyl group in the N^ε^-amino group of the lysine residues, eliminating the positive charge of this amino acid and by that may alter the function of proteins by influencing their catalytic activity, their ability to interact with other proteins or their subcellular localisation.^44^ Also, acetylation is found at *N*-terminal regions of proteins affecting protein stability and subcellular localisation.^59^ This modification was first described for histones’ *N*-terminal domains where it regulates chromatin structure and gene transcription, but with the development of more sensitive proteomic equipment, thousands of non-histone proteins have been identified acetylated in prokaryotes and eukaryotes, affecting a wide variety of cellular pathways.^44^ This is also true for trypanosomatids, where acetylation is present in proteins regulating metabolism, oxidative stress response and other key cellular processes.^48^ ^,^ ^49^ ^,^ ^60^ ^,^ ^61^ ^,^ ^62^ Lysine acetyltransferases (KATs) are responsible for transferring the acetyl group from acetyl-CoA to the lysine residues, while the lysine deacetylases (KDACs) remove the acetyl groups.^63^ ^,^ ^64^ Moreover, bromodomain-containing proteins (BRDs) bind acetylated lysines and recruit proteins that will perform downstream regulatory functions.^65^ All this regulatory machinery is present in *Leishmania*, but compared to higher eukaryotes, as humans, it is reduced and generally diverged, which make them excellent targets for chemical inhibitors.^62^ ^,^ ^66^ The *Leishmania* acetylation regulatory machinery is formed by seven, six and seven, KDACs, KATs and BRDs coding genes, respectively.^66^ ^,^ ^67^ The seven KDACs are divided in two classes: zinc-dependent lysine deacetylases (DACs), with four genes, and NAD^+^-dependent or sirtuins with three genes; while two KATs families, MYST (four genes) and GNAT (two genes) are found.^66^ Most of this machinery has been characterised in *Leishmania*, except the members of KDACs from the DAC family and the members of GNAT family of KATs. Although the scenario to explore KATs, KDACs and BDRs as drug targets in *Leishmania* is favorable, with several genes found to be essential for parasite survival, efforts have been focused on the sirtuin proteins (Sir2rp1-3), which had been proved to be crucial for both *in vitro* and *in vivo* growth of *Leishmania*.^68^ ^,^ ^69^ ^,^ ^70^ Two of the three sirtuins, Sir2rp1 and rp2, have been characterised as essential for the parasite and involved in cytoskeleton and mitochondrial metabolism, respectively.^68^ ^,^ ^69^ ^,^ ^70^ Indeed, in the past few years they have been targeted by several inhibitors with the aim of identifying potential antileishmanial agents.^70^ ^,^ ^71^ ^,^ ^72^ Recently, Jones et al., have characterised BDFs (bromodomain factors or proteins containing bromodomains) from *L. mexicana* and found that five of seven are essential for the parasite and that BRD5 is important for chromatin structure regulation.^73^ The BDFs have been explored in the last years as drug targets for treatment of different diseases such as cancer, with several clinical phase I and II studies underway,^74^ which could contribute to explore the *Leishmania* BDFs by drug repositioning. Moreover, the availability of different BDF protein structure will help to accelerate this process.^67^

Considerable progress has been made in the last decades at unveiling KDACs and bromodomain proteins (BDPs) as promising therapeutic targets in *Leishmania*.We still have a great opportunity to better explore the regulatory machinery of protein acetylation for the development of new antileishmanial molecules by characterising the other members and also by testing the myriad of available KATs, KDACs and BDRs inhibitors using high content drug screenings.

*Protein crotonylation* - Lysine is by far the most modified amino acid with many different acylation marks, been reported to be present in the N^ε^-amino group of this residue, including succinylation, malonylation and crotonylation.^75^ ^,^ ^76^ Many of these modifications have been identified in trypanosomatids, distributed in histone and non-histone proteins.^49^ Crotonylation is added in the lysine by crotonyltransferases and removed by decrotonylases. Several studies demonstrated that KATs and KDACs might act as crotonyltransferases and decrotonylases, respectively.^77^ ^,^ ^78^ ^,^ ^79^ ^,^ ^80^ However, unlike acetylation, the YEATS (Yaf9, ENL, AF9, Taf14, Sas5) domain-containing proteins are the reader modules identified to bind lysine-crotonylated residues.^81^ These proteins have been described as members of several complexes involved in gene expression regulation, DNA repair and chromatin structure remodeling.^81^ ^,^ ^82^ ^,^ ^83^ Two YEATS containing-domain genes have been identified in the *Leishmania* genome,^84^ which is half of the members found in humans.^85^ Based on protein sequence comparison, they have been designated Yaf9 and ENL and preliminary data from our group suggest that they are essential genes for *L. mexicana* procyclic form (unpublished data). The role of YEATS in pathogenic organisms is still poorly explored, but recent studies from *Candida albicans* YEATS observed that mutant knockout cell lines for the two genes encoding YEATS had an impairment growth and a significant reduction in virulence *in vivo* infection assays using murine models,^86^ pointing to the importance of these proteins for pathogenicity of infectious agents. Initial screenings have been performed to identify chemical molecules with potential to inhibit the YEATS domain-containing protein from humans and few compounds have been described with great potential to be explored in the future in *Leishmania*.^87^ ^,^ ^88^ ^,^ ^89^ However, it remains difficult to affect robust cellular responses by targeting these domains and one reason could be the challenge of displacing a multidomain complex by antagonising a single reader domain.

*Other modifications* - Recent studies have demonstrated the importance of arginine methylation and ubiquitination for *Leishmania* biology. Arginine methylation is catalysed by PRMTs (Protein Arginine Methyltransferases) by the transfer of a methyl group from S-adenosyl methionine to the target arginine residue, which increases their hydrophobicity.^90^ *Leishmania* has seven PRMTs members (PRMT1-7) and it has been reported that PRMT7 could impact parasite infectivity in the mouse model animal and the RNA-binding capacity of target-proteins.^91^ ^,^ ^92^ Ubiquitination is characterised by the attachment of one or more ubiquitin proteins to substrates and is essential for the regulation of a variety of cellular processes, such as protein degradation. Addition of ubiquitin molecules is carried out by the sequential actions of E1 ubiquitin-activating (E1), E2 ubiquitin-conjugating (E2) and E3 ubiquitin ligase (E3) enzymes; while the reverse process is promoted by de-ubiquitinating proteins (DUBs).^43^ ^,^ ^93^ Two works by Damianou et al., and Burge et al., recently demonstrated the importance of DUBs and ubiquitin-ligases in *Leishmania* stage differentiation using different biochemical and molecular approaches, validating some of the members of these families as potential drug targets in the parasite.^94^ ^,^ ^95^ These findings expand the possibilities to explore the PTMs regulatory machinery as a drug target in *Leishmania*.

Concluding remarks

Current main challenges on drug discovery and development converges to standardise pre-clinical protocols worldwide and meet the requirements for a product market fit aligned with the pharmaceutical industry and specific features of the suffering areas such as drug resistance, cold chain (temperature stability) and price. Metallodrugs deserve more attention as antileishmanial entities and collaborative drug discovery is strongly encouraged. Advances have been made on elucidating PTMs as crucial mechanisms to maintain homeostasis being key to regulate the cellular mechanisms involved in the *Leishmania* adaptation between its two hosts. Progress in the field has unraveled many of these modifications and how they modulate crucial biological processes in trypanosomatids, but we still miss a lot of information regarding this in *Leishmania*. Thus, identifying the set of proteins that are modified and the PTMs present in *Leishmania*, as well as, the proper characterisation of the regulatory machinery of these PTMs will increase substantially the number of potential drug targets to be explored. Redox homeostasis can be considered a target for drug discovery and elucidation. In this case PTM on cysteine residues can produce distinct alterations and alter cell signaling mediated by redox-sensitive proteins and drug pharmacology.^96^ The impact of PTM on cancer pharmacology has been recently demonstrated by molecular simulations were regulation of High Mobility Group Box (HMGB) proteins bound to platinated DNA (PtDNA) lesions can increase complex stability towards greater affinity for PtDNA resulting in cisplantin sensitisation.^97^ Inspired by these discoveries, advancing drug target elucidation and drug development in a collaborative manner, will certainly reduce time on finding alternative drugs to tackle leishmaniasis. None of these will serve without consortium and massive investments for clinical trials. In this regard, pharmaceutical industry and governments are fundamental to close the cycle and advance on fighting NTDs.
